# Flux-Aided Synthesis of Lu_2_O_3_ and Lu_2_O_3_:Eu—Single Crystal Structure, Morphology Control and Radioluminescence Efficiency

**DOI:** 10.3390/ma7107059

**Published:** 2014-10-20

**Authors:** Justyna Zeler, Lucjan B. Jerzykiewicz, Eugeniusz Zych

**Affiliations:** 1 Faculty of Chemistry, University of Wroclaw, 14. F. Joliot-Curie Street, 50-383 Wroclaw, Poland; E-Mails: justyna.zeler@chem.uni.wroc.pl (J.Z.); lucjan.jerzykiewicz@chem.uni.wroc.pl (L.B.J.); 2 Wroclaw Research Centre EIT+, 147 Stablowicka Street, 54-066 Wroclaw, Poland

**Keywords:** lutetium oxide, flux synthesis, single crystal structure, radioluminescence

## Abstract

Li_2_SO_4_ or (Li_2_SO_4_ + SiO_2_)-mixture fluxes were used to prepare a Lu_2_O_3_:Eu powder phosphor as well as an undoped Lu_2_O_3_ utilizing commercial lutetia and europia as starting reagents. SEM images showed that the fabricated powders were non-agglomerated and the particles sizes varied from single microns to tens of micrometers depending largely on the flux composition rather than the oxide(s)-to-flux ratio. In the presence of SiO_2_ in the flux, certain grains grew up to 300–400 μm. The lack of agglomeration and the large sizes of crystallites allowed making single crystal structural measurements and analysis on an undoped Lu_2_O_3_ obtained by means of the flux technique. The cubic structure with *a* = 10.393(2) Å, and Ia3 space group at 298 K was determined. The most efficient radioluminescence of Lu_2_O_3_:Eu powders reached 95%–105% of the commercial Gd_2_O_2_S:Eu.

## 1. Introduction

At the end of 20th century, the Lu_2_O_3_:Eu X-ray phosphor became a subject of thorough research [1,2,3,4,5,6,7,8,9,10,11,12,13,14,15,16,17,18,19]. Also, other activators were used, especially for lasing in Lu_2_O_3_ sintered transparent bodies [20,21]. With its impressive density of 9.84 g/cm^3^, high effective Z-number (63.7) and light yield similar to Gd_2_O_2_S-based phosphors [3,22,23,24] both lutetia powders and sintered ceramics were considered attractive for X-ray scintillator detectors [3,22,25]. Furthermore, with an energy band gap of about 5.85 eV [25], Lu_2_O_3_:Eu was recognized to have potential to produce as much as about 75,000 ph/MeV upon ionizing radiation excitation. This is well beyond the performance reported in literature [25], which gives hope that there still is room for significant improvement. The extraordinary absorption coefficient of X-rays by photoelectric effect rather than Compton scattering [26,27] allows using thinner Lu_2_O_3_:Eu phosphor layers, which translates into reduced light scattering, *i.e.*, image smudging [27]. Clearly, Lu_2_O_3_:Eu may offer important advantages as X-ray phosphor if only its light yield could be improved and morphology well controlled.

High quality screens, with uniformly and densely packed grains, require spherical, rather uniform in size particles. It was shown that the spherical size of particles allows reducing the screen thickness by 1/3 [17,28]. Also, effective sintering of powders into translucent or transparent bodies, especially using pressureless techniques, is very much dependent on the starting powder morphology and its thermal history [17,29,30,31,32]. Yet, literature data on controlling the Lu_2_O_3_:Eu powders morphology are only scant [33,34,35,36] and those that are available were never confronted with the phosphors performance under X-rays. With this paper we try to partially fill this gap. We thus shall report on the evolution of morphology of Lu_2_O_3_:Eu powders produced using Li_2_SO_4_/(Li_2_SO_4_ + SiO_2_)-flux-aided preparation technique. Since this fabrication procedure gave occasionally crystallites with sizes sufficient for single crystal structure determination, we took advantage of that and preformed such analysis. Up to now, the only direct determination of Lu_2_O_3_ structure was performed on single crystals obtained by a micro-pulling down (μ-PD) technique as well as a laser heated pedestal growth (LHPG) [37].

## 2. Results and Discussion

### 2.1. Structural Analysis

**Figure 1:**
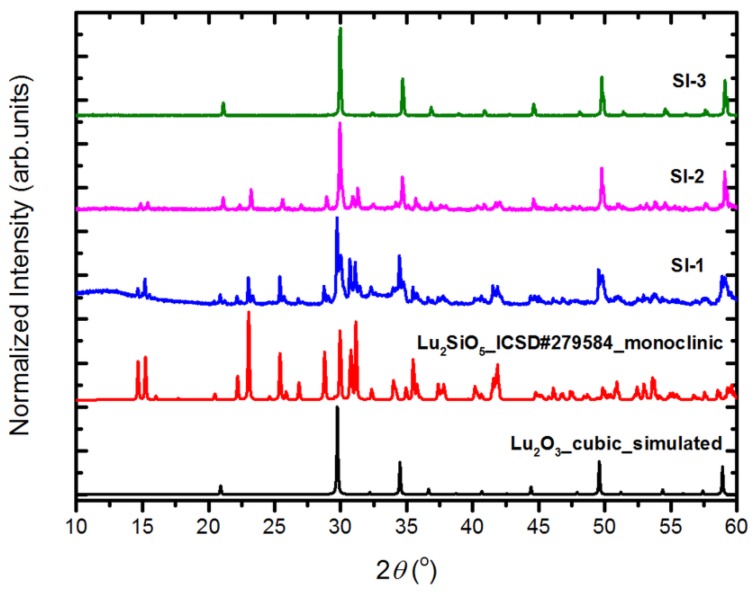
The measured XRD patterns of the powders of Series I: SI-1, SI-2, SI-3, together with simulated pattern of a cubic Lu_2_O_3_ (data from this work) and the pattern of a monoclinic Lu_2_SiO_5_.

Let us start by presenting the X-ray diffraction (XRD) patterns of powders of Series I (Figure 1), which supposedly should/might provide Lu_2_SiO_5_ (LSO) powders, as the lutetia and silica were mixed in 1:1 molar ratio. Indeed, when the synthesis was only 1-h long (sample SI-1) a main fraction of the product was LSO, with Lu_2_O_3_ present as an impurity, readily detected but not a massive fraction. In the case of the sample heated for 2 h (SI-2), the powder was already largely composed of Lu_2_O_3_, while LSO existed only as an impurity phase. A 20-h prolonged heating at 1300 °C (SI-3) ended up with a single phase cubic Lu_2_O_3_ powder without any traces of LSO, as seen by the XRD technique. Hence, the XRD data of powders of Series I proves, that in the presence of significant amount of Li_2_SO_4_-flux, silica is effectively eliminated from the batch upon prolonged heating at elevated temperatures. Additional experiments, results of which we do not present here, proved that the process of removal of SiO_2_ from the reacting mixture of Lu_2_O_3_ and SiO_2_ was faster with increasing Li_2_SO_4_ flux content and with increasing temperature—at 900 °C it was much slower, while at 1400 °C it was even faster than at 1300 °C. This we took into account by proceeding with synthesis of samples of Series II–IV. The structural measurements of all powders of Series II–IV prove that each of the products is cubic Lu_2_O_3_. Yet, we noted that prolonged washing with hot water to remove the Li_2_SO_4_ flux may end up with slight contamination by LuO(OH). This should be taken into account when recovering the lutetia powder from the flux. Heat treatment at 900 °C for 2 h effectively eliminated the LuO(OH), and only Lu_2_O_3_ was seen in XRD patterns.

**Figure 2:**
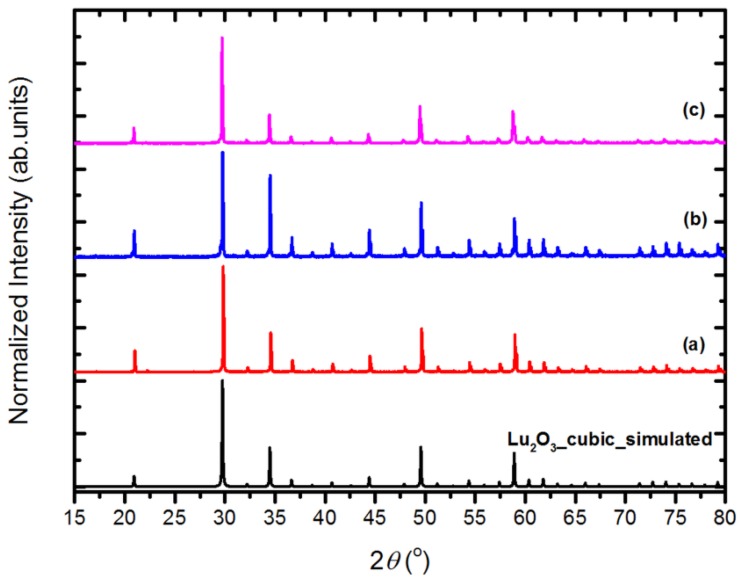
Representative XRD patterns of Lu_2_O_3_ powders of Series II, III and IV. (**a**) SII-1; (**b**) SIII-3; (**c**) SIV-2, together with simulated pattern of a cubic Lu_2_O_3_ (data from this work).

In Figure 2, we present selected XRDs of samples of Series II–IV. They are representative of the other powders of the three series. Also, the powders of Series III, in which the most important change compared to Series II was addition of some SiO_2_ to the flux, contain only cubic Lu_2_O_3_, and no silica or LSO was detected. Addition of 5 mol% of Eu_2_O_3_ to the reacting mixture (Series IV) did not affect the crystallization process and the product was a solid solution of Lu_2_O_3_:Eu. In this case, the diffraction lines were slightly shifted towards smaller angles due to the larger size of Eu^3+^ ion (0.947 Å) compared to Lu^3+^ (0.861 Å). Hence, already the structural measurements indicated that Eu_2_O_3_ dissolved in the Lu_2_O_3_ host during the synthesis process giving activated Lu_2_O_3_:Eu phosphor.

### 2.2. Flux-Stimulated Evolution of the Powders Morphology

**Figure 3:**
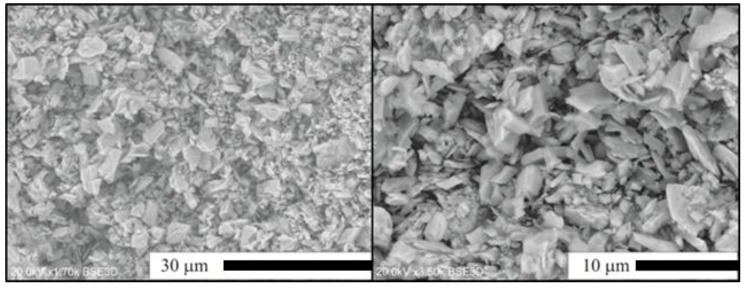
SEM images of the Lu_2_O_3_ starting materials (Stanford Materials Corporation).

**Figure 4:**
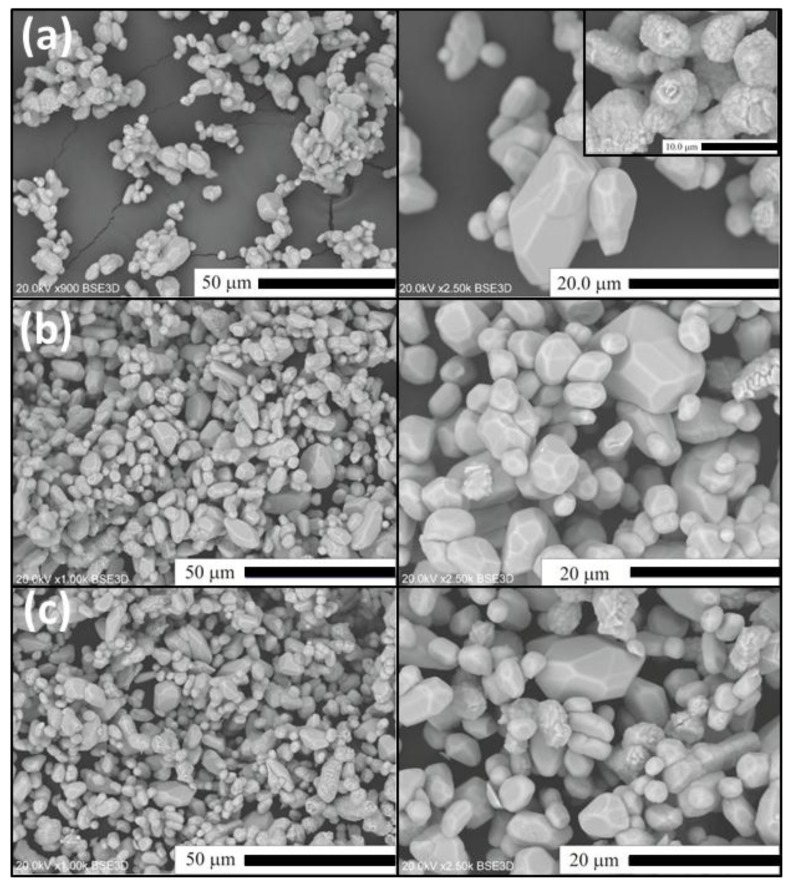
SEM images of Lu_2_O_3_ were prepared using Series II synthesis for (**a**) SII-1; (**b**) SII-2; (**c**) SII-3.

In this section, we shall present and discuss differences in the morphology of Lu_2_O_3_ powders of the Series II–IV as well as evolution of the morphology within each series. For comparison, in Figure 3 we show scanning electron microscope (SEM) images of Lu_2_O_3_ starting material used in our synthesis processes. As we shall see, the morphology of the raw lutetia is much different from all morphology of the powders we synthesized using the flux. Figure 4 presents SEM images of Lu_2_O_3_ powders of Series II in which the flux consisted of Li_2_SO_4_ exclusively, and was used in different proportions to the Lu_2_O_3_. Clearly, alteration of the ratio of the flux against Lu_2_O_3_ did not affect the morphology to any significant degree. Basically, all powders of Series II consist of grains of similar sizes of 3–4 μm, though the largest of them are of about 20 μm in diameter. The grains are not very uniform when size is taken into account. The grains are mostly monocrystalline and form polyhedra of regular and similar shapes. Some granules consist of aggregated smaller crystallites (see the upper top image). These, though not very numerous, were seen in each specimen of Series II.

**Figure 5:**
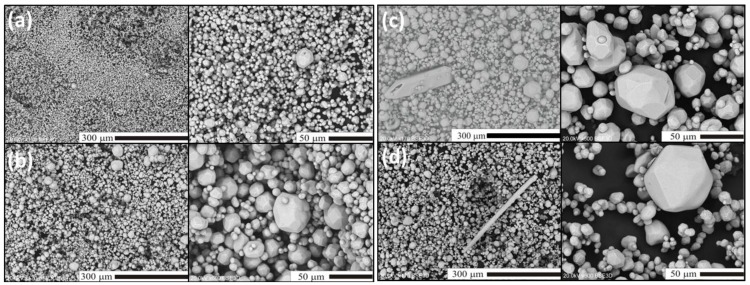
SEM images of Lu_2_O_3_ of samples of Series III. (**a**) SIII-1; (**b**) SIII-2; (**c**) SIII-3; (**d**) SIII-4. Note that some grans in SIII-3 and SIII-4 powders have the unexpected shape of needles.

In Series III (Figure 5), when synthesized using a mixture of Li_2_SO_4_ and SiO_2_ as the flux, the grains become more uniform in size, compared to Series II. For the SIII-1 powder the sizes are typically 3–6 μm and only occasionally 20 μm grains are observed (Figure 5a). In the case of sample SIII-2 (Figure 5b), when the content of SiO_2_ in the flux (compared to SIII-1) was roughly tripled, the grains become clearly larger. A six-fold increase of the SiO_2_ in the flux (compared to SIII-1) further increases, though not significantly, the fraction of the largest grains (Figure 5c), among which we can easily select 70–100 μm monocrystals. Rarely, needle-shaped grains are observed as presented in Figure 5c. The needles are as long as about 200–300 μm. Their fraction gets larger with an increasing amount of the flux compared to Lu_2_O_3_ (compare Figure 5c,d). Since for cubic structures the needle-like shape of monocrystalline grains is unexpected, we decided to perform a full structural analysis of such a crystal to unambiguously resolve if Lu_2_O_3_ can crystallize in a different structure in the conditions we applied for the preparations. The results will be presented in the next section.

**Figure 6:**
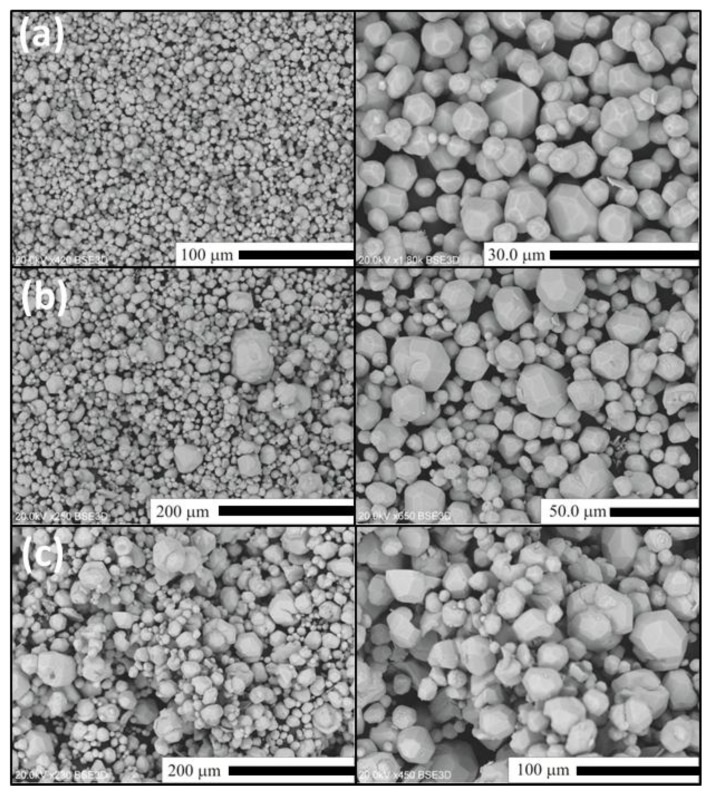
SEM images of Lu_2_O_3_:Eu of samples of Series IV. (**a**) SIV-1; (**b**) SIV-2; (**c**) SIV-3.

We already mentioned when discussing XRD patterns that by using a mixture of Lu_2_O_3_ and Eu_2_O_3_ it was possible to produce a Lu_2_O_3_:Eu phosphor by means of the Li_2_SO_4_-SiO_2_ flux technique. The morphology of the material (see Figure 6) was not much different from its undoped counterparts presented above. Interestingly, it was routinely indicated by inductively coupled plasma (ICP) analysis that only about 60%–65% of Eu indeed entered the lutetia host during the flux-aided synthesis.

### 2.3. Single Crystal Structural Analysis

**Figure 7:**
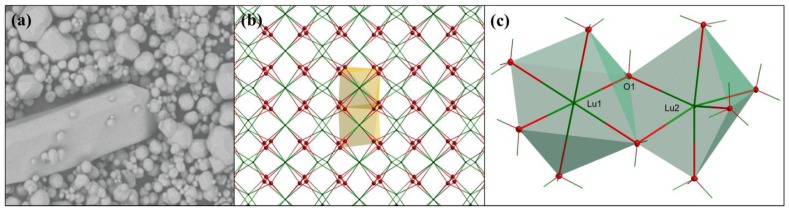
(**a**) The grain of the powder SIII-3 selected for the monocrystalline structural measurements; (**b**) a packing diagram of Lu_2_O_3_. For clarity, only two polyhedra were shown; (**c**) coordination environment of Lu1 and Lu2 in Lu_2_O_3_.

As we already saw, see Figure 5c,d, in some cases needle-shaped crystals could be selected within a batch. This observation encouraged us to verify the crystal structure of both the needles and the more regular, polyhedral particles. Both types of crystals gave practically the same results and here we shall present those for the needle-shaped crystal selected from the powder SIII-3 (see Figure 5c and Figure 7a). Below a detailed description of the results is given. It was unambiguously confirmed that this Lu_2_O_3_ crystal, despite its specific shape, crystallized in a cubic system, the space group is Ia 3 with *a* = 10.393(2) Å, *V* = 1122.6(6) Å^3^, *Z* = 16, *T* = 298 K. Other crystal data are listed in Table 1, together with refinement details. Complete crystallographic data for the structural analysis have been deposited with the Fachinformationszentium Karlsruhe (FIZ), CSD No. 428012. Further details of the crystal structure investigations may be obtained from Fachinformationszentrum Karlsruhe, Eggenstein-Leopoldshafen, Germany [38] on quoting the appropriate CSD number. The cubic cell parameter calculated using powder XRD measured for sample SIII-3 gave = 10.4216(9) Å. This is in good agreement with the value obtained for single crystal measurements.

**Table 1:** Crystal data and structure refinement for Lu_2_O_3_.

Chemical Formula	Lu_2_O_3_
Formula Mass	397.94
Crystal system	regular
*a*/Å	10.393(2)
Unit cell volume/Å^3^	1122.6(6)
Temperature/K	298(2)
Space group	Ia3
No. of formula units per unit cell, *Z*	16
No. of reflections measured	8753
No. of independent reflections	1789
*R*_int_	0.0425
Final *R*_1_ values (*I* > 2σ(*I*))	0.0287
Final *wR*(*F*^2^) values (*I* > 2σ(*I*))	0.0555
Final *R*_1_ values (all data)	0.0307
Final *wR*(*F*^2^) values (all data)	0.0560

The Lu_2_O_3_ represents the rare-earth sequioxide C-type of structure, isostructural to Y_2_O_3_. Figure 7c shows that the crystal structure of Lu_2_O_3_ offers two different positions for the metal ion (Lu1 and Lu2). Lutetium atoms are connected by bridging O1 and O1^i^ oxygen atoms. Coordination numbers (*CN*) of both Lu1 and Lu2 are *CN* = 6. Lu1 occupies a perfectly centrosymmetric position (C_3i_) in the lattice with all Lu1-O1with symmetry codes: (i) −*y* + 1/2, −*z* + 1/2, −*x* + 1/2; (ii) *y*, *z*, *x*; (iii) −*x* + 1/2, −*y* + 1/2, −*z* + 1/2; (iv) −*z* + 1/2, −*x* + 1/2, −*y* + 1/2; (v) *z*, *x*, *y*; distances equal 2.2392(18) Å. Lu2 atom possesses non-centrosymmetric C_2_ local site symmetry in the cubic structure of Lu_2_O_3_. The population of the Lu1 is 25% and the fraction of Lu2 is 75% of all metal sites. Figure 7b shows the packing of Lu_2_O_3_. Two types of layers of Lu sites are present. One of them contains only Lu2 atoms showing the non-centrosymmetric geometry (C_2_), while the other consists of the same number of Lu1 (C_3i_) and Lu2 (C_2_) ions. The crystal structure of Lu_2_O_3_ is stabilized by bridging oxygen atoms between lutetium atoms. Table 2 presents all geometric parameters of the structure.

**Table 2:** Geometric parameters (Å, °).

Atoms involved	Distance (Å), Angle (°)
Lu1—O1	2.2392(18)
Lu1—Lu2	3.4395(7)
Lu2—O1	2.2277(19)
Lu2—O1^i^	2.2945(19)
Lu2—O1^x^	2.1954(18)
O1—Lu1—O1^i^	80.10(6)
O1—Lu1—O1^ii^	99.90(6)
O1—Lu1—O1^iii^	180.0
O1^x^—Lu2—O1^xi^	87.14(10)
O1^x^—Lu2—O1	99.66(6)
O1^xi^—Lu2—O1	109.94(8)
O1—Lu2—O1^xii^	138.89(9)
O1^x^—Lu2—O1^i^	79.57(7)
O1^xi^—Lu2—O1^i^	165.12(5)
O1—Lu2—O1^i^	79.15(9)
O1^x^—Lu2—O1^xiii^	165.12(5)
O1—Lu2—O1^xiii^	78.91(6)
O1^i^—Lu2—O1^xiii^	114.36(9)

Symmetry codes: (i) −*y* + 1/2, −*z* + 1/2, −*x* + 1/2; (ii) *y*, *z*, *x*; (iii) −*x* + 1/2, −*y* + 1/2, −*z* + 1/2; (x) −*z* + 1, −*x* + 1/2, *y*; (xi) −*z* + 1, *x*−1/2, −*y* + 1/2; (xii) *x*, −*y*, −*z* + 1/2; (xiii) −*y* + 1/2, *z* − 1/2, *x*.

The results presented here are generally very similar to the recently published data on the single crystal structure of Lu_2_O_3_ [37]. It may be surprising that for our crystal made with the Li_2_SO_4_ flux the final *R_1_* (*I* > 2σ(*I*)) value is even smaller than for the two crystals reported in [37], which were made by a μ-PD and LHPG techniques. The unit cell of the one obtained by the μ-PD showed a slightly smaller size of *a* = 10.364(2) Å compared to that fabricated by LHPG, which was *a* = 10.403(2) Å. The difference was suggested to result from O-vacancies supposedly present in the slightly yellow μ-PD crystal formed in a reducing atmosphere. The size of the unit cell obtained for our crystal made by the flux technique is much closer (10.393(2) Å) to the size of the LHPG crystal, and is almost identical with the size of the unit cell obtained for sintered ceramics (10.39120(6) Å) [37].

### 2.4. Radioluminescent Properties

**Figure 8:**
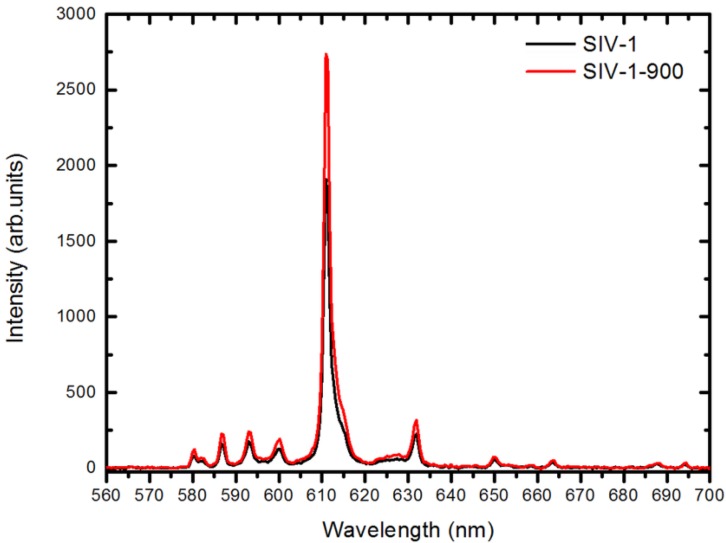
XEL emission spectra of the sample SIV-1 (black line) and SIV-1-900 (red line). See also Table 3.

As we mentioned in the Introduction, Lu_2_O_3_:Eu arouses interest mostly for its radioluminescent properties. Thus, having the possibility to control the morphology of its powders, we also were interested in testing their performance under excitation with X-rays. Figure 8 shows the X-ray excited luminescence (XEL) spectra of Lu_2_O_3_:Eu powders of Series IV. The spectral distribution of the emitted photons is typical for Lu_2_O_3_:Eu, with the main luminescence located at 610 nm and resulting from the ^5^D_0_→^7^F_2_ transition. The overall XEL efficiencies of Lu_2_O_3_:Eu were related to the performance of a commercial Gd_2_O_2_S:Eu (GOS:Eu) measured at the same conditions. All as-made samples performed similarly and on average their light yield reached 70%–80% of the GOS:Eu efficiency. Thus, the phosphor performance was not affected by the particle sizes to any significant degree. When the raw powders were additionally heated at 900 °C (SIV-1-900 and SIV-2-900) and 1200 °C (SIV-1-1200), the XEL efficiencies increased to 95%–105% of the commercial phosphor (see Figure 8 and Table 3). An increase in the formal concentration of Eu from 5% to 8% (samples SIV-3 *versus* SIV-4) in raw Lu_2_O_3_:Eu did not further affect the phosphor performance. Since Lu_2_O_3_:Eu can be used in layers when at least 1.5-fold thinner—due to its higher absorption of X-rays than Gd_2_O_2_S:Eu—it provides some advantage over GOS. On the other hand, its significantly higher price reduces its competitiveness. Hence, only a further enhancement of the luminescence efficiency under X-rays might make Lu_2_O_3_:Eu a real competitor for GOS:Eu. While using the (Li_2_SO_4_-SiO_2_) flux allows improving the phosphor morphology, the XEL efficiency, though high, is still too low to beat up the effective and reasonably priced GOS.

**Table 3:** XEL efficiency of Lu_2_O_3_:Eu *vs.* commercially offered Gd_2_O_2_S:Eu.

Lu_2_O_3_:Eu sample #	XEL Efficiency (%)
SIV-1	72
SIV-1-900	105
SIV-1-1200	97
SIV-2	66
SIV-2-900	95
SIV-3	80
SIV-4	75

## 3. Experimental Section

Lu_2_O_3_ and Eu-doped Lu_2_O_3_ powders were prepared by means of a Li_2_SO_4_ or (Li_2_SO_4_ + SiO_2_) flux (Li_2_SO_4_, Alfa Aesar, 99.7%, Karlsruhe, Germany; SiO_2_, Sigma Aldrich, ~99%, St. Louis, MO, USA) method using a mixture of commercially offered Lu_2_O_3_ (Stanford Materials Corporation, 99.995%, Aliso Viejo, CA, USA) and Eu_2_O_3_ (Stanford Materials Corporation, 99.999%). Four series of compounds were prepared and details are given in Table 4. Powders of series I–III contain no activator. Taking advantage of the knowledge on the results of Series I–III, fourth Eu-activated powders, Lu_2_O_3_:Eu (5 mol%), were prepared using mixtures of Li_2_SO_4_ and SiO_2_ as the flux (see Table 4 for details). These products gave Series IV of powders. To document the influence of Li_2_SO_4_ flux on the continuous removing of SiO_2_ from the reacting mixture, three samples (Series I) were prepared using the molar ratio of Lu_2_O_3_:SiO_2_ = 1:1, as in the Lu_2_SiO_5_, see Table 4 for other details. In typical synthesis, the mixture of the reagents and the flux was transferred to a Pt-Ir (90%–10%) crucible which was next heated up at 1400 °C for 50 h in air. In the case of Series I, the temperature was lowered to 1300 °C and the time varied from 1 up to 20 h. After heating, the mixture was cooled down to room temperature (RT). To recover the product, the Li_2_SO_4_ flux was washed out with hot water and finally the powder was dried at ~80 °C in air for a few hours. For radioluminescent measurements, fractions of samples SIV-1 and SIV-2 were additionally heated at 900 °C and 1200 °C to see how this treatment affects their performance. These samples are named SIV-1-900 and SIV-2-900.

The powder X-ray diffraction patterns were measured using a D8 Advance Diffractometer from Bruker (Billerica, MA, USA) with Ni-filtered CuK_α_ radiation (λ = 1.540596 Å) in the range of 2θ = 10°–100°, and with the step of 2θ = 0.008°. Single crystal X-ray diffraction data for undoped Lu_2_O_3_ were collected at room temperature using the ω-scan technique on Xcalibur diffractometer (Agilent Technologies, Santa Clara, CA, USA) equipped with Ruby CCD-detector using graphite-monochromatized MoKα radiation (λ = 0.71073 Å) [39]. The data were corrected for Lorentz-polarization effects and for absorption. The structure was refined with the full-matrix least-squares procedure on F^2^ by SHELXL [40] on coordinates of atoms were taken from a previously reported isomorphous crystal of Y_2_O_3_. All atoms were refined anisotropically. The products’ morphology was tested by means of SEM imaging with Hitachi S-3400N scanning electron microscope (Hitachi High-Technologies, Tokyo, Japan) equipped with an energy dispersive X-ray spectroscopy (EDX) EDAX analyzer. The room temperature X-ray excited luminescence—measurements of the Eu-activated powders of Series IV were performed using white radiation from a Cu X-ray tube working under the voltage of 40 kV and a current of 10 mA. The generated emission photons were collected with a 74-UV lens connected to a QP600-2-SR-BX waveguide which transferred the luminescent light to an Ocean Optics HR2000CG-UV-NIR Spectrometer (Ocean Optics, Dunedin, FL, USA). Efficiency of the XEL was estimated using a commercially offered powder of Gd_2_O_2_S:Eu as the benchmark kindly supplied by Phosphor Technology. The real content of Eu in Lu_2_O_3_:Eu powders was determined by means of Inductively Coupled Plasma (ICP) technique using an ARL 3410 ICP Spectrometer (Fisons Instruments, Ecublens, Switzerland).

**Table 4:** Exemplary amounts of starting materials for the four series of synthesized powders.

Series	Sample #	Batch Composition (g)				Process Parameters	Product Composition
		Lu_2_O_3_	Eu_2_O_3_	Li_2_SO_4_	SiO_2_	Temperature (°C)/Time (h)	
I	SI-1	1.7585	–	13.97	0.2661	1300/1	Lu_2_SiO_5_, Lu_2_O_3_
	SI-2	1.7585	–	13.97	0.2661	1300/2	Lu_2_SiO_5_, Lu_2_O_3_
	SI-3	1.7585	–	13.97	0.2661	1300/20	Lu_2_O_3_
II	SII-1	2	–	2	–	1400/50	Lu_2_O_3_
	SII-2	2	–	4.77	–		Lu_2_O_3_
	SII-3	2	–	9.54	–		Lu_2_O_3_
III	SIII-1	2	–	4.77	0.045		Lu_2_O_3_
	SIII-2	2	–	4.77	0.15		Lu_2_O_3_
	SIII-3	2	–	4.77	0.3		Lu_2_O_3_
	SIII-4	2	–	9.54	0.3		Lu_2_O_3_
IV	SIV-1	1.9	0.0884	4.77	0.045		Lu_2_O_3_
	SIV-2	1.9	0.0884	9.54	0.3		Lu_2_O_3_
	SIV-3	1.9	0.0884	4.77	0.3		Lu_2_O_3_
	SIV-4	1.84	0.1415	4.77	0.3		Lu_2_O_3_

## 4. Conclusions

In this paper, we showed that Li_2_SO_4_-flux-aided synthesis of Lu_2_O_3_ and Lu_2_O_3_:Eu gives non-agglomerated powders with particles whose sizes may be controlled to some extent by addition of some SiO_2_ to the flux. No traces of silica were detected in the final product. Large enough single crystals could be selected to perform structural analysis. This confirmed that lutetia is isostructural with cubic yttria and *a* = 10.393(2) Å. This is in agreement with very recently published data on Lu_2_O_3_ structure determined on single crystals made by μ-PD and LHPG techniques. X-ray excited luminescence spectra were typical for Lu_2_O_3_:Eu and the light yield reached 95%–105% of the yield of commercial GOS:Eu, which is not enough to compete effectively with this phosphor in practical applications.
